# Cofilin dysregulation alters actin turnover in frataxin-deficient neurons

**DOI:** 10.1038/s41598-020-62050-7

**Published:** 2020-03-23

**Authors:** Diana C. Muñoz-Lasso, Belén Mollá, Pablo Calap-Quintana, José Luis García-Giménez, Federico V. Pallardo, Francesc Palau, Pilar Gonzalez-Cabo

**Affiliations:** 1 CIBER de Enfermedades Raras (CIBERER), Valencia, Spain; 2 Department of Physiology, Faculty of Medicine and Dentistry. University of Valencia-INCLIVA, Valencia, 46010 Spain; 3 Associated Unit for Rare Diseases INCLIVA-CIPF, Valencia, Spain; 4 Instituto de Biomedicina de Valencia (IBV), CSIC, Valencia, 46010 Spain; 5 Institut de Recerca Sant Joan de Déu and Department of Genetic & Molecular Medicine and IPER, Hospital Sant Joan de Déu, Barcelona, 08950 Spain; 6 Hospital Clínic and Division of Pediatrics, University of Barcelona School of Medicine and Health Sciences, Barcelona, Spain

**Keywords:** Molecular neuroscience, Peripheral neuropathies

## Abstract

Abnormalities in actin cytoskeleton have been linked to Friedreich’s ataxia (FRDA), an inherited peripheral neuropathy characterised by an early loss of neurons in dorsal root ganglia (DRG) among other clinical symptoms. Despite all efforts to date, we still do not fully understand the molecular events that contribute to the lack of sensory neurons in FRDA. We studied the adult neuronal growth cone (GC) at the cellular and molecular level to decipher the connection between frataxin and actin cytoskeleton in DRG neurons of the well-characterised YG8R Friedreich’s ataxia mouse model. Immunofluorescence studies in primary cultures of DRG from YG8R mice showed neurons with fewer and smaller GCs than controls, associated with an inhibition of neurite growth. In frataxin-deficient neurons, we also observed an increase in the filamentous (F)-actin/monomeric (G)-actin ratio (F/G-actin ratio) in axons and GCs linked to dysregulation of two crucial modulators of filamentous actin turnover, cofilin-1 and the actin-related protein (ARP) 2/3 complex. We show how the activation of cofilin is due to the increase in chronophin (CIN), a cofilin-activating phosphatase. Thus cofilin emerges, for the first time, as a link between frataxin deficiency and actin cytoskeleton alterations.

## Introduction

Friedreich’s ataxia (FRDA) patients display sensory axonal neuropathy characterised by slowly dying-back degeneration of the dorsal root ganglia (DRG) with a selective lack of proprioceptive neurons^1,2^. This neurodegeneration continues with axonal degeneration of posterior columns, spinocerebellar and corticospinal tracts, and myelinated fibres in the central nervous system (CNS)^3^.

In 96% of cases, the mutation that causes FRDA is a pathological homozygous expansion of an intronic GAA triplet repeat in the gene codifying for a small mitochondrial protein, frataxin (FXN)^4^. As a consequence of this mutation, frataxin expression is drastically reduced in all tissues, especially in the heart and peripheral nerves^5,6^. In cells, frataxin is necessary for iron-sulfur cluster synthesis and antioxidant defence (reviewed in^7^). Furthermore, frataxin deficiency has been frequently associated with an increase in reactive oxygen species (ROS) (reviewed in^8^), generated by alterations in the electron transport chain and iron accumulation in mitochondria (reviewed in^9,10^).

The increase in ROS impairs the cellular redox balance, which causes the oxidation and glutathionylation of cytoskeletal proteins such as actin and tubulin. Cytoskeletal protein alterations have been associated with axonal neuropathy in several neurodegenerative diseases, including FRDA. Earlier research in fibroblasts from FRDA patients showed how frataxin deficiency affects intermediate filaments by reducing the proper distribution of vimentin along with cytoskeleton structures^11^. Later, Pastore *et al*. showed how an increase in oxidative stress in these cells increases actin S-glutathionylation, producing a decreased polymerisation of globular actin monomers (G-actin) and therefore reducing the level of filamentous actin (F-actin)^12^. Immunohistochemical studies of the spinal cord of FRDA patients necropsies displayed an increase in tubulin tyrosination and increased tubulin S-glutathionylation in motor neurons^13^. Additional studies of microtubule dynamics in frataxin-silenced motoneurons showed how an increase in tubulin glutathionylation reduces its polymerisation and neurite growth^14^.

DRG neurons have a unique intrinsic capacity to grow and to regenerate their axons even after being damaged^15,16^. Neurite growth is guided by the growth cone (GC) until it reaches its target tissue. The GC is a highly dynamic structure enriched in actin and microtubules that needs to be continuously supplied with proteins and energy, which are mostly provided by mitochondria^17–19^. The cellular process responsible for the regulated transfer of mitochondria, synaptic vesicles, lipids and various organelles from the cell body to nerve terminals (anterograde transport) or in the opposite direction (retrograde transport) is axonal transport (reviewed in^20^). Within the GC, actin is continually reorganised in actin filaments (F-actin) by constant cycles of polymerisation/depolymerisation. The dynamics of actin change the morphology of the GC, extending, retracting or pausing growth. Several cytoskeletal proteins and enzymes responsible for regulating F-actin polymerisation are calcium- or ATP-regulated proteins (reviewed in^21,22^). Here we investigate the effects of the absence of frataxin on the structure and dynamics of the GCs of DRG sensory neurons in the YG8R mouse model. The YG8R mouse model contains the human mutation present in most FRDA patients and is, therefore, one of the most suitable models to study the pathophysiology of FRDA^23^. This model exhibits slow dying-back neurodegeneration starting at the peripheral nerve roots of DRGs^24^, cytoskeletal disorganization, mitochondrial dysfunction caused by mitochondrial depolarization, the increase in ROS, energetic failure, and improper calcium handling^25^ that can be reversed by the use of phosphodiesterase inhibitors^26^.

We found that aberrant changes in the morphology of GCs were associated with an increase in the conversion of G-to F-actin (F/G-actin ratio) in sensory neurons of YG8R mice. These results correlated with hyperactivation of cofilin due to the increased levels of the phosphatase chronophin, and the actin-related protein (ARP)2/3 complex. These alterations in actin cytoskeleton and dysregulation of actin-binding proteins might explain the reduced neurite growth of frataxin-deficient DRG neurons.

## Results

### Changes in the morphology of the growth cones of frataxin-deficient neurons in adult dorsal root ganglia from the YG8R mouse

Under normal conditions, adult DRG neurons exhibit a unique capacity to grow and to regenerate their neurites after being damaged. The actin cytoskeleton plays a vital role in this process. As previously described^27^, we have confirmed that the length of the longest neurite of isolated DRGs neurons from YG8R mice (224.9 ± 11.33 µm) was shorter than in control animals (249.7 ± 10.75 µm) (Fig. S1). This data supports the idea that the role of frataxin is relevant to neurite growth in DRG neurons and probably to a proper function of the GCs, allowing neurites to grow and regenerate.

**Figure 1:**
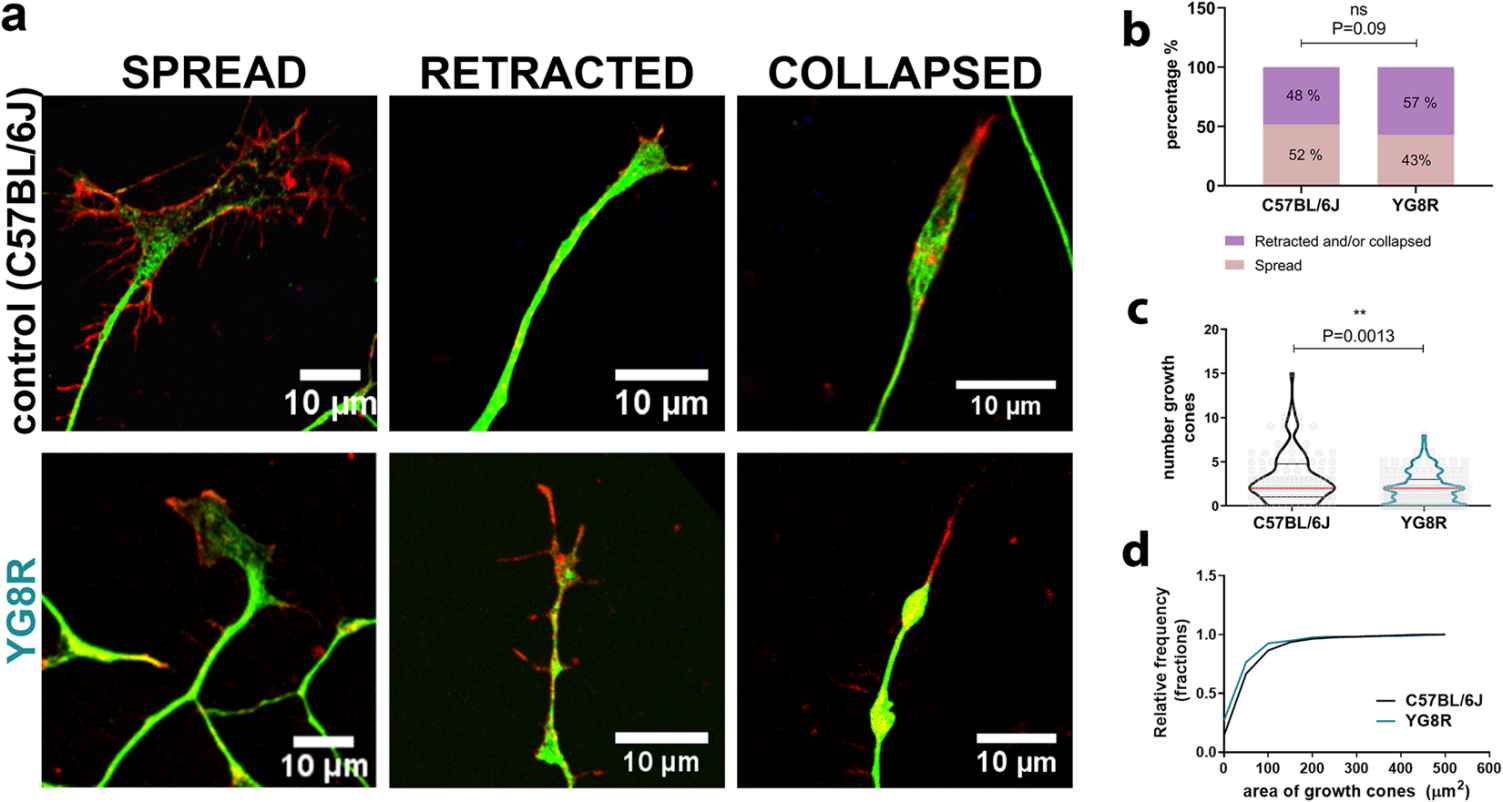
Growth cones of frataxin-deficient neurons exhibit morphological alterations. (**a**) Representative images of different types of growth cones of YG8R (lower panel) and control mice (C57BL/6J) (upper panel). Growth cones were visually classified as spread or collapsed/retracted according to characteristic β-tubulin-III (green) and F-actin (red) distribution. (**b**) Distribution of the growth cones in the spread or collapsed/retracted between both genotypes. Differences between genotypes were assessed using Chi-square test: P = 0.09. (**c**) Sensory neurons from YG8R mice exhibited fewer growth cones per neuron compared to controls. The data distribution is presented with violin plots. Data (grey dots), the median (red horizontal lines) and interquartile (black horizontal lines) are shown. Mann-Whitney test was used to analyse significant changes between genotypes: **P = 0.0013. (**d**) Cumulative distribution of the growth cone area was plotted for both genotypes. Most of the growth cones from YG8R mice were smaller (0–100 µm^2^) than those from the controls. Mann-Whitney test was used to analyse significant changes between genotypes: **P = 0.0058.

Neurite growth is a cellular process entirely dependent on the correct function of the GCs. GCs are located at the most distal part of growing neurites and, together with Schwann cells, allow the axon to innervate their target tissue properly. The morphology of GCs is directly related to their functionality^28^, which can be determined by studying their molecular structure. Based on the structure of microtubules and filamentous actin, three different types of GCs have been described previously in the literature: spread-GCs, which reflect an ongoing growth; retracted-GCs, which indicate a paused increase; and collapsed-GCs, which indicate a neurite retraction^29^. We identified these GCs, by immunofluorescence, using this classification to determine if the morphology was affected in frataxin-deficient neurons of adult DRG from the YG8R mouse (Fig. 1a). The retracted or collapsed GCs can be only distinguished when unpolymerized tubulin is detected^29^, therefore we included both retracted and collapsed GCs in the same group (Fig. 1b). We observed an increasing trend of retracted or collapsed GCs and a reduction in the number of spread GCs in frataxin-deficient neurons compared with control neurons (P-value = 0.09), which may suggest that the reduced neurite growth previously observed is associated with a change in the morphology of their GCs.

To further investigate this observation, we analysed the number and size of GCs in cultured DRG neurons by immunodetection of β-tubulin III. The number of GCs in each neuron (Fig. 1c) was significantly lower in the YG8R genotype (2.01 ± 0.17) than in control mice (3.17 ± 0.27; P = 0.0013). Regarding size, 92.6% of the GCs in the frataxin-deficient neurons had a reduced area (between 0–100 µm^2^) compared to 86.5% of the GCs in control neurons, indicating that the GCs from YG8R neurons are smaller (60.81 ± 4.99) than those from control neurons (73.19 ± 5.12; P-value = 0.006) (Fig. 1d).

### Growth cones of frataxin-deficient neurons exhibit an abnormal increase in the F/G actin ratio

**Figure 2:**
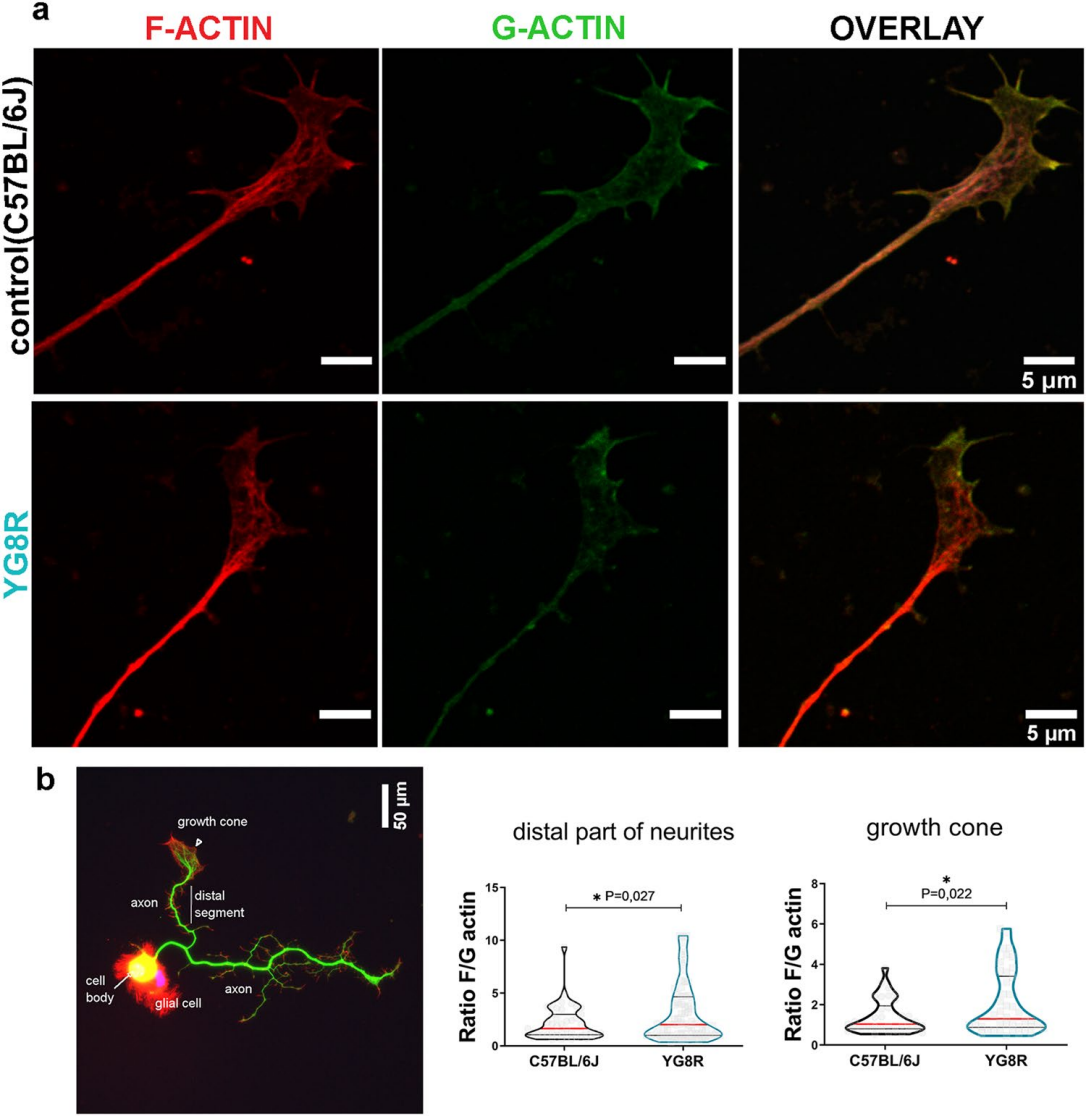
Growth cones of frataxin-deficient neurons of YG8R mice contain high levels of F- and G-actin. (**a**) Representative images of growth cones of sensory neurons from dorsal root ganglia (DRG) stained with the phalloidin-TRITC and Deoxyribonuclease I probes to detect endogenous levels of F actin (red) and G-actin (green) respectively. Confocal images show the maximal intensity projection. Merge of images show colocalization regions of F- and G-actin, as well as the differences in the expression and distribution of F/G-actin in growth cones. Scale bars 5 µm. The F/G-actin ratio was quantified for the distal segment (most distal part of neurites) and growth cones (**b**). The image illustrates the three major compartments (cell body, neurites and growth cones) of a growing-adult DRG neuron (24 hours in culture). Rhodamine, anti-β-Tubulin III and DAPI were used to detect F-actin (red), microtubules (green) and nuclei (blue). A white line surrounds the distal segment that has been used in the study to analyse the levels of F- and G-actin. The ratio was significantly increased in the distal segment (b, middle) and the growth cones (b, right) of YG8R mice compared to the controls (C57BL/6J). Violin plot shows the distribution of the values for the ratio in both genotypes. Red horizontal bars indicate the median, black horizontal bars indicate quartiles and grey dots shows the values. Welch’s t-test was used to analyse significant changes between genotypes: *P ≤ 0.05.

A suitable function of GC relies on the cytoskeleton, particularly on actin. Actin is synthesised in higher amounts in the GC than in any other neuronal compartment^30^. The polymerisation of globular actin (G- actin) produces filamentous actin (F-actin) and actin arcs, which are the structural base of the peripheral and transitional domains of GCs, respectively^31–33^. Therefore, the balance between F-actin and G-actin in the GC, known as the F/G-actin ratio, reflects the state of the F-actin turnover, necessary for a proper function of GCs and an optimal neurite growth (reviewed in^34^). Here, we studied the F/G-actin ratio in two compartments of frataxin-deficient DRG neurons: the GCs and the distal segment (approximately 50 µm^2^ of the longest neurite starting at the base of GC). For the GCs, we have chosen those that fulfil the parameters of a SPREAD morphology because it is the state when the actin polymerisation is highly active. Primary cultures of DRGs from controls and YG8R were cultured for 48 hours and stained with Deoxyribonuclease I-Alexa Fluor 488 and phalloidin-TRITC probes to identify G- and F- actin respectively. After measuring fluorescence intensity by confocal microscopy (Fig. 2), we found that the average F/G-actin ratio was significantly increased in the GCs with a SPREAD morphology of frataxin-deficient neurons from YG8R mice (2.06 ± 0. 24) compared to the controls neurons from C57BL/6J mice (1.40 ± 0.14; P ≤ 0.05; P = 0.022) (Fig. 2b). The ratio was also significantly increased in the distal segment of the neurites (YG8R: 3.1 ± 0.33; control: 2.17 ± 0.25; P = 0.027) (Fig. 2b). The distribution of F-actin in the GCs of YG8R neurons compared with that observed in the control neurons is characterised by the absence of the concentration at the leading edge of the GCs (Fig. 2a) which usually coincides with an absence of G-actin and a higher concentration of F-actin in the central region of the GCs with a disorganised architecture. These results indicate the disequilibrium in F-/G-actin homeostasis in frataxin-deficient neurons. The increase in F-actin levels suggests a disruption in actin dynamics that would explain the morphological changes in the GCs and the reduced neurite growth observed in DRG neurons from the YG8R mice.

### Frataxin deficiency causes the hyperactivation of Cofilin and ARP2/3 complex

Actin-binding proteins (ABPs) regulate the continuous polymerisation and depolymerization of actin filaments. To go deeper into molecular mechanisms that could explain the increase in the F/G-actin ratio in axons and GCs in DRG neurons from the YG8R mouse, we tested the expression levels of two crucial ABPs involved in this molecular process: cofilin-1 (COF1) and the ARP2/3 complex. These two ABPs are essential proteins involved in the F-actin turnover and branching along actin filaments (reviewed in^35^). COF1 is the major isoform in non-muscle tissues and belongs to the family of ADF/cofilin that disassembles F-actin. COF1 binds to F-actin and destabilizes and breaks the filaments producing the release of free monomers of actin (G-actin). In contrast to COF1, the ARP2/3 complex is responsible for building new branches or short filaments of actin. Since COF1 is the factor that severs actin filaments and ARP2/3 promotes the creation of new short filaments, the expression levels of these two proteins could explain the dysregulation of the F/G-actin ratio in the cells and consequently, overall changes in the GCs.

**Figure 3:**
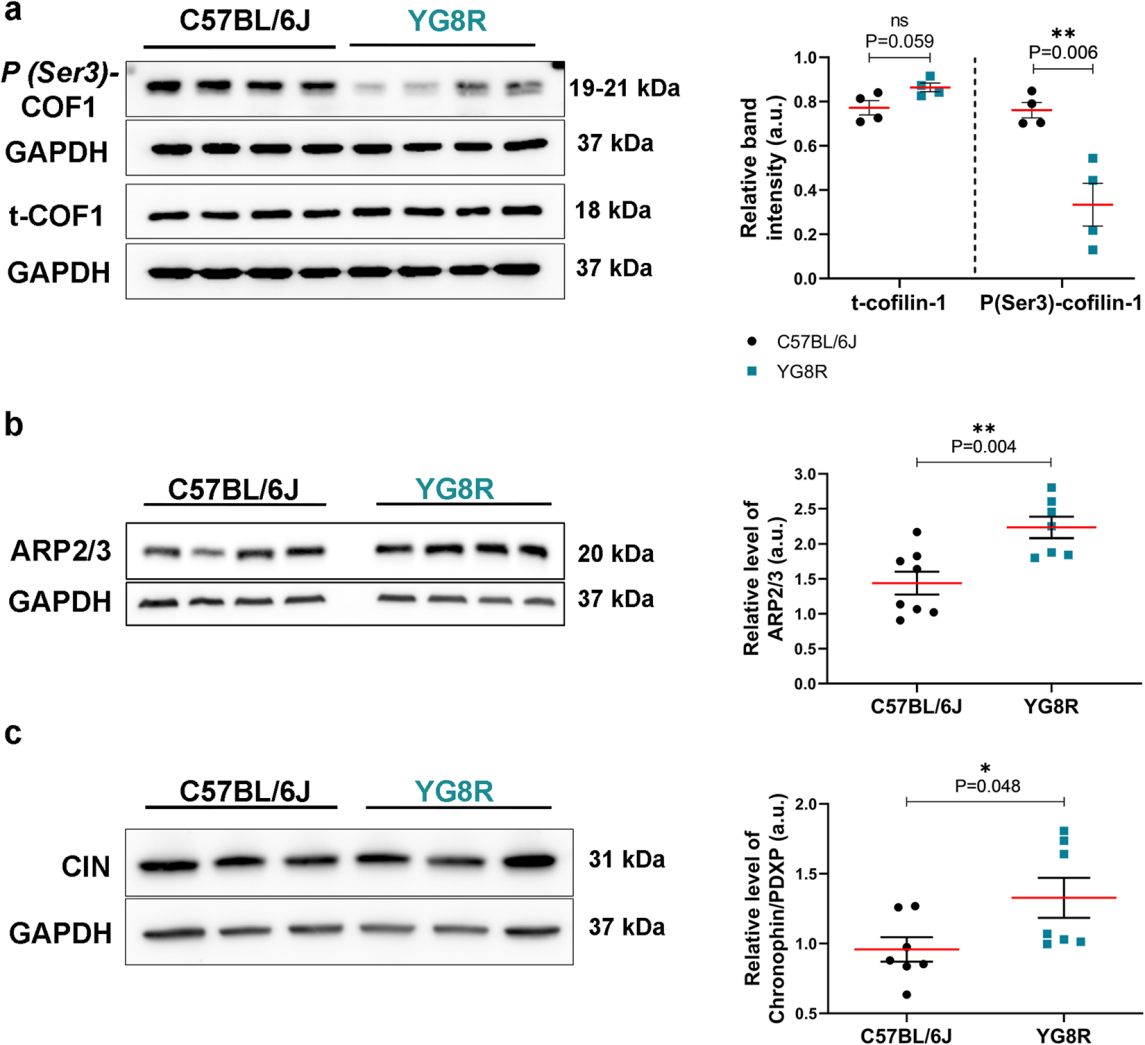
Frataxin deficiency in sensory neurons of YG8R mice modulates the activity of cofilin, ARP2/3 complex and the phosphatase Chronophin. Western blots and quantitative analysis of the expression of total cofilin (active form) and phosphor (Ser3)-cofilin (inactive form) (**a**), the ARP2/3 complex (**b**) and the phosphatase Chronophin (CIN) (**c**) in dorsal nerve roots of YG8R and control mice (C57BL/6J). Total cofilin exhibited an increase in dorsal nerve roots of YG8R mice compared with control mice (C57BL/6J), whereas the P(Ser3)-cofilin expression was reduced significantly, suggesting an increase in cofilin activity (n = 4 per genotype). In addition, there was a significant increase in the expression of ARP2/3 complex in dorsal nerve roots of YG8R mice (n = 8) compared to controls (C57BL/6J, n = 7). The expression of the phosphatase CIN also resulted in a significant increase in dorsal nerve roots of YG8R mice (YG8R, n = 8; Control, n = 8). The red horizontal bar indicates the mean. All the values are plotted with ± s.e.m. Quantitative Significant changes between genotypes were assessed using Student’s t-test. *P ≤ 0.05, **P ≤ 0.01. Full-length blots are presented in Supplementary Fig. 3.

We analysed the expression level of COF1 (Fig. 3a) and ARP2/3 complex (Fig. 3b) by Western blot in the dorsal nerve roots of controls and YG8R mice. The results showed a significant decrease in the expression of the inactive form of COF1 (phospho-(Ser3)-cofilin-1) in the YG8R mice (n = 4, 0.33 ± 0.10) compared to controls (n = 4, 0.76 ± 0.04; P = 0.006). This was associated with an increase in the active form of COF1 (n = 4, 0.86 ± 0.02) compared to controls (n = 4, 0.77 ± 0.03) (Fig. 3a). Besides the increase in activated COF1 levels, the ARP2/3 complex levels were also significantly increased in the YG8R mice (n = 7, 2.23 ± 0.15) compared with controls (n = 8, 1.44 ± 0.16; P = 0.004) (Fig. 3b). Therefore, the changes previously observed in the F/G-actin ratio in distal segments and GCs could be the result of the COF1 activated form, and the increase in the levels of the ARP2/3 can be interpreted as a neuronal response to maintain the balance of the two forms of actin levels (G- and F-actin) in the axons and the GCs.

### Upstream regulators of COF1 are dysregulated in the frataxin-deficient neurons from YG8R mouse

The activity of COF1 is mainly modulated by its phosphorylation (inactivation)/dephosphorylation (activation) in the serine 3 (Ser3) residue. This process is catalysed mainly by the serine protein LIM (Lim11, Isl-1, and Mec-3) kinase 1 (LIMK1). In contrast, two phosphatases are known to catalyse dephosphorylation: slingshot (SSH) phosphatase and PDXP/chronophin (CIN). In order to understand how COF1 expression is modulated by frataxin deficiency, we tested the expression levels of the upstream regulatory proteins of COF1. The expression level analysis of total LIMK1 and its active form (Phospho-(Thr508)-LIMK1) showed no changes in the frataxin-deficient dorsal nerve roots of YG8R mouse (Fig. S2a). However, total CIN levels in dorsal nerve roots from YG8R mice were significantly higher (n = 7, 1.33 ± 0.14) than in controls (n = 7, 0.96 ± 0.09; P = 0.048) (Fig. 3c), partially explaining the lower levels of the phosphorylation of COF1.

### The dysregulation of COF1 and the ARP2/3 complex does not alter the expression of other actin-binding proteins

Among other ABPs are profilin (PFN), the Wislott-Aldrich Syndrome protein (WASp) and DAAM-1, which have a synergic action with COF1 and ARP2/3. Profilin sequesters the ADP-G actin subunits released by COF1 activity and speeds up the conversion to ATP-G-actin. There is evidence that ARP2/3 competes with DAAM1 to use the G-actin coupled to PFN^36^. WASp stimulates the actin-nucleating activity of ARP2/3 complex, and once activated, this complex can bind to pre-formed filaments and imitate growing ends that allow the polymerisation of free monomers and therefore the assembly of new short filaments. Our results did not show significant changes in the expression of WASp, PFN, and DAAM1 in the frataxin-deficient dorsal nerve roots of YG8R mouse (Fig. S2b,c).

## Discussion

Cytoskeletal abnormalities have been proposed to contribute to dying-back neurodegeneration in FRDA^10,12,14,24^. Nevertheless, the consequences of these abnormalities have been poorly explored in the most critical neuronal target tissue of FRDA, the dorsal root ganglia (DRG). In this work, we used DRG neurons of YG8R mice, a well-characterized FRDA model, to show how two essential proteins that regulate the actin cytoskeleton, cofilin and the ARP2/3 are unbalanced, affecting essential structures of DRG neurons such as the GC.

The GC allows neurons to form the neurites and the axon. It is located at the most distal part of a growing neurite and axon and has a unique morphology and dynamics. The dynamics of GCs rely on the continuous reorganization of actin filaments and microtubules (MTs)^37^, which is coordinated by complex Ca^2+^-regulated signalling pathways. In our study, we observed a significant reduction in the number and area of GCs in frataxin-deficient DRG neurons from YG8R mice. Furthermore, most of these GCs exhibited a retracted or collapsed morphology instead of the spread morphology, which corresponds to the active state of GCs. These novel findings might explain the inhibition of neurite growth observed in DRG primary cultures from YG8R mice in this work as well as by other authors^14,27^.

The equilibrium between F and G-actin marks the stability of actin filaments in axons and mediates the response of the GCs to external signals of the GCs^38^. At the molecular level, in the GCs of the frataxin-deficient neurites from YG8R mice, the F/G-actin ratio was increased, suggesting an alteration of the normal turnover of actin filaments. Previously, Pastore *et al*. investigated the levels of G-and F-actin in fibroblasts from FRDA patients^12^. Contrary to our results in GCs of DRG neurons, in fibroblasts from FRDA patients, they found a reduced signal of F-actin as a consequence of an increased glutathionylation of actin, which reduces its ability to be polymerized^12^. These conflicting results may be explained by the difference in morphology between a fibroblast and a neuron, which influences the dynamics of actin.

In this work, we investigated the molecular mechanisms that cause the increase in F/G-actin ratio in the GCs of the YG8R DRG neurons. Our results show a significant reduction in the inactive form of cofilin-1 (P-Ser-3), suggesting a hyperactivation of cofilin-1. This phenomenon is associated with an increase in the expression of one crucial ABP, the actin-related protein (ARP) 2/3- complex, perhaps as a response to the increased activity of cofilin. It is known that there is a synergistic effect between cofilin and ARP2/3^39–41^. When cofilin-1 is active (non-phosphorylated) it severs old ADP-F-actin, and then ARP2/3 binds to new ATP-F-actin to generate barbed ends^41^. These lead to polymerization of new F-actin, also favoured by the increase in the levels of G-actin, and later it ages into old F-actin. Our results show an altered F/G-actin ratio, both in the growth cone and in the most distal area of the axon. The fact that in the axons of the neurons of the dorsal ganglion (dorsal root) we found an increase in the levels of ARP2/3 and hyperactivation of cofilin-1 makes us wonder if these molecular alterations could be the cause of the changes in the F/G-actin ratio. New studies analysing the variation of these proteins address to the growth cone will be necessary to determine their involvement in growth cone dynamics fully.

Cofilin-1 is a pH-sensitive F-actin depolymerizing protein^42^, essential for the neuronal development^43^, required for growth cone extension^44,45^, axonal transport^46^, and is involved in cell death signalling^47,48^. The state of phosphorylation of cofilin-1 (inactivation) is regulated by three mechanisms consisting on the regulation of the physiological levels of ATP^49^, the oxidative stress^49–52^, and the cytoplasmic Ca^2+^ levels (reviewed in^53^). These three processes are affected in the frataxin-deficient DRG neurons from YG8R mice^25^ and are known to induce the activation of phosphatases chronophin (CIN) that dephosphorylates cofilin-1. It is well described that the hyperactivation of cofilin-1 has other unhealthy consequences, such as the formation of pathogenic inclusions in axons known as actin rods^49–51^. Actin rods result from the linking of cofilin (non-phosphorylated) with ADP-actin and the generation of disulfide bonds between the cofilin-1 molecules (reviewed in^54^). Actin rod formation impairs cofilin1-mediated severing of F-actin preventing actin turnover. Two factors promote the formation of the actin rods: oxidative stress by forming intermolecular disulphide bonds between the cofilin-1 cysteines^55^; and ATP depletion by increasing ADP-actin levels and activating a cofilin phosphatase, chronophin CIN^49^. In our model, we observed an increase in CIN levels, which in an ATP depletion environment explains the decreased levels of phosphorylated cofilin. The significant pathological consequences of actin rods are the blocking of intracellular trafficking, abnormal distribution of cellular organelles, such as mitochondria, and synaptic loss^56^. Therefore, actin rod formation could contribute to the axonal sensory neuropathy observed in frataxin-deficient DRG neurons from the YG8R mouse by blocking axonal transport, as we have demonstrated previously^24,25^.

In this work, we demonstrate the dysregulation of the actin cytoskeleton in frataxin-deficient neurites of DRG neurons from the YG8R mice as a result of hyperactivation of cofilin-1 and the complex ARP2/3, which could affect the dynamics of growth cones and neurite growth. Recent work has determined that cofilin hyperactivation is age-dependent^57^, so it would be interesting to analyze these results at the embryonic stage. Assessing if there is a dysregulation of cofilin that could contribute to a failure in the neurite growth of embryonic neurons would help to understand the hypoplastic phenomena previously described in FRDA patients^1^. As a whole, our results show for the first time an imbalance in the activity of cofilin that could explain, at least partially, the neuropathy of FRDA. Future research will determine if cofilin is a potential molecular target for a therapeutic approach in FRDA.

## Material and Methods

### Animals

YG8R mice were purchased from The Jackson Laboratory Repository (stock no. 008398. Maine, US). Animals were maintained and selected from a colony of YG8RxYG8R as previously described^24^. We used hemizygous mice (YG8R), containing one allele of the mutant FXN transgene with the lowest level of FXN, and the C57BL/6J as control mice (control). All handling and protocols were carried out following the practices established and approved by the Bioethics Subcommittee of *Consejo Superior de Investigaciones Científicas* (CSIC). All the mice were euthanized by cervical dislocation at age 24 months.

### Primary-culture of adult neurons from the dorsal root ganglia

C57BL/6J and YG8R mice were euthanized by cervical dislocation following the principals for the care and use of laboratory animals. The whole dorsal root ganglia (DRG) were dissected from the entire length of the vertebral column of each mouse strain (control: C57BL/6J; mouse model: YG8R) at 24 months of age. Immediately, DRG capsules containing the cell bodies were separated from the dorsal nerve roots and maintained in L-15 (Leibovitz) medium. Then, DRGs capsules were incubated with collagenase and with 2.5% (v/v) trypsin, then washed in Ham’s Nutrient Mixture F12 (Sigma-Aldrich), followed by gentle mechanical trituration using a P1000 pipette in 1 ml of complete medium [Ham’s F12 medium supplemented with 2 mM L-glutamine (Sigma-Aldrich); 35% (w/v) Albumax (InvitrogenTM); 16 µg/ml putrescine; 60 ng/ml progesterone; 400 ng/ml-thyroxine; 38 ng/ml sodium selenite; 340 ng/ml triiodothyronine; 60 µg/ml penicillin; 100 µg/ml streptomycin]. Then, isolated neurons were seeded in 24 well dishes containing cover glasses (13 mm), previously coated with 0.5 mg/ml of poly-DL-Ornitin and 0.01 mg/ml of laminin (Sigma-Aldrich), with a density of 1000 neurons/mm^2^. Neurons were cultured with 1 ml of medium and supplemented with neurotrophins [mNGF; BDNF; NT3 (Peprotech bioNova); 10 ng/ml of each].

### Immunofluorescence for the study of the morphology of growth cones

Isolated neurons from DRGs were cultured for 24 hours in order to analyse entire neurons with their growth cones. Primary cultures were fixed carefully in 4% (w/v) paraformaldehyde supplemented with 4% (w/v) saccharose at room temperature for 15 minutes and washed carefully with PBS-1X three times. The blocking and permeating solution [10% (v/v) Fetal Bovine Serum (FBS); 0,1% (v/v) Triton x-100] was added and allowed to act for 30 minutes at room temperature. Primary antibodies were diluted in blocking and permeating solution and then incubated at 4 °C within a wet chamber in darkness overnight. Cultures were washed three times with PBS-1X leaving for 5 minutes between washes. A secondary antibody to detect β-tubulin-III (Alexa Fluor 488 Conjugate, Thermo Fisher Scientific D12371) and probe to detect F-actin (Phalloidin-TRITC, Sigma-Aldrich, P1951) were diluted in blocking and permeating solution and then incubated at room temperature within a wet chamber in darkness for 1 hour. Cover glasses were mounted with DAPI-Fluoromount-G (Southern Biotech). A total of 108 neurons from three biological replicates (mice n = 3) were analysed for each genotype (YG8R, control). GCs were selected randomly and later classified into two groups: a) spread and b) collapsed or retracted according to the arrangement of F-actin and microtubules. For this selection, we settled some criteria based on the previous description in the literature^29,58^. Growth cones were classified such as SPREAD growth cones when they were positive at least for three characteristics: (i) A flat shape at the most distal part of the neurite; (ii) Microtubules appear as a tight straight bundle; (iii) Microfilaments are concentrated in the growth cone. Growth cones were classified such as COLLAPSED OR RETRACTED growth cones when they exhibited the following characteristics (i, ii in combination with one or more of the other characteristics): (i) A bulb at the most distal part of the neurite; (ii) Enlarged distal región; (iii) A thin trailing remnant; (iv) Few filopodia and lamellipodia in the growth cone; (v) Bending of the microtubules in the growth cone; (vi) Redistribution of the microfilaments in the growth cone; (vii) Microtubules are reconfigured into coiled and sinusoidal bundles; (viii) Microfilaments are concentrated in the most distal region of the distal region. For this analysis, at least 176 growth cones from individual neurons were evaluated for each genotype. The number of GCs per neuron was obtained by visual inspection of confocal images of neurons stained for β-tubulin-III and F-actin.

### Measurements of the area of growth cones

Confocal images were acquired from neurons stained for β-tubulin-III and F-actin in a representative position of Z-axis with confocal microscope Leica TCS SP8 with HC PL APO CS2 40x/1.30 OIL objectives. Images were then processed with the software Fiji/ImageJ (N.I.H). Area of GCs was obtained with the LAS AF-Advanced Fluorescence software (Leica). The most significant GC of each neuron was manually selected following the filopodia outline. For area, a total of 171 (n = 3, control) and 175 (n = 3, YG8R) growth cones from individual neurons were evaluated from three biological replicates for each genotype.

### Study of the fluorescence of globular (G) and filamentous (F) actin

Isolated neurons from DRGs were cultured for 48 hours and then carefully fixed and blocked as described above. Neurons were incubated simultaneously with the probes to detect F- [1:200, Phalloidin-TRITC] and G-actin [1: 200, Deoxyribonuclease I (DNAseI), Alexa Fluor 488 Conjugate, Thermo Fisher Scientific D12371]. Fluorescently labelled DNAseI specifically and sensitively detects G-actin in cells fixed in formaldehyde and permeabilised in detergent^59^. As a consequence, we have fixed the neurons in 4% paraformaldehyde and permeabilization with Triton x100 (which is considered a comparatively mild detergent, non-denaturing). This method has been used by other works to evaluate F/G actin ratios in neuron-like cells^38^. The probes were diluted in the same blocking solution and were incubated at room temperature in a wet chamber and darkness for 1 hour. All cover glasses were mounted with DAPI-Fluoromount-G (Southern Biotech). For this analysis, active growth cones were selected by choosing those growth cones that fulfilled the characteristics of a SPREAD morphology as outlined above.

In order to quantify F- and G-actin into GCs, the filopodium outline was manually selected. To get measurements for the neurites, an area of approximately 50 µm^2^ was manually selected starting at the base of the GC (herein denominated distal segment). Images were acquired with a Leica TCS SP8 confocal microscope and with an HC PL APO CS2 40x/1.30 OIL objective. The maximum projection was obtained from Z-series (10–15 sections and a step of 0,29 µm). The sum of pixels was selected as a parameter for fluorescence intensity (RawIntDen). A total of 34 (n = 3, control) and 42 (n = 3, YG8R) GCs and axonal segments from individual neurons were evaluated from three biological replicates. The ratio was calculated for each GC and axonal segment dividing intensity values obtained for F-actin by those obtained for G actin.

### Analysis of proteins by western blotting

Approximately 50 DRGs and their dorsal nerve roots (which contains axons) were dissected from the whole spinal cord of each mouse strain (control: C57BL/6J; mouse model: YG8R) at 24 months of age. Immediately, dorsal nerve roots were separated from the cell bodies and were independently frozen in liquid nitrogen and stored at −80 °C until further processing.

Total protein extracts from dorsal nerve roots were obtained by resuspending tissues in 200 µl of ice-cold lysis buffer [50 mM Tris-HCl pH 7.4; 1% (v/v) Triton X-100; 1.5 mM MgCl, 50 mM NaF, 5 mM EDTA, 1 mM sodium orthovanadate, 0,1 mM PMSF, 1 mM DTT, protease and phosphatase inhibitors cocktails (Sigma-Aldrich)]. Immediately, tissues were mechanically homogenized simultaneously with TissueLyser II (QIAGEN) through high-speed shaking in plastic tubes with stainless steel (diameter 5 mm). Five cycles of 50 Hz for 30 seconds were applied with 30 seconds in between each cycle. Then, protein lysates from tissues were centrifuged at 14,000 rpm for 15 minutes at 4 °C, and the supernatant containing whole protein extracts were collected and quantified with Bradford protein assay (Bio-Rad).

For SDS-PAGE electrophoresis, protein extracts were resuspended in loading buffer 5X[250 mM Tris-HCl pH 6.8; 10% (w/v) SDS; 0,5% (w/v) Bromophenol blue; 50% (v/v) glycerol and 500 mM DTT]. 40 µg of total protein extract per sample was denatured by boiling for 5 minutes before loading into the Miniprotean Electrophoresis System (BioRad). Proteins were loaded in 12% (w/v) polyacrylamide gels and run with Tris-Glycine running buffer [25 mM Tris; 192 mM Glycine and 0.1% (w/v) SDS]. To test Cofilin (active or inactive) and ARP2/3, proteins were transferred from the gels to nitrocellulose membrane (GE Healthcare). To test chronophin (CIN), proteins were transferred from the gels to a Polyvinylidene Fluoride (PVDF) membrane with constant voltage (100 V) for 1 hour at 4 °C with transfer buffer [25 mM Tris; 192 mM Glycine and 20% (v/v) Methanol]. Membranes were incubated for 1 hour with appropriate blocking solution (phosphorylated proteins: 5% (w/v) BSA in TBS-T [20 mM Tris-HCl pH 7.5; 150 mM NaCl; 0.1% (v/v) Tween-20]; for other proteins: 5% skimmed milk in TBS-T).

Membranes were incubated with primary antibodies to detect total-cofilin (1:1000, ab54532), phosphorylated cofilin (1:1000, sc-12912), complex ARP2/3 (1:500, Millipore MABT95), Chronophin (1:1000, Cell Sig. #4686) and GAPDH (1:1000, GAPDH sc32233) diluted in the appropriate blocking solution overnight at 4 °C with agitation. Membranes were washed three times with TBS-T for 10 minutes and were incubated with the respective secondary antibody conjugated with peroxidase enzyme for 1 hour at room temperature with agitation (1:5000, Goat Anti-Mouse IgG, H & L Chain Specific, 401211, Sigma Aldrich; 1:5000, Anti-mouse IgG, HRP-linked Antibody, Cell Sig. # 7076). Proteins bands were detected using chemiluminescence using the ECL Plus Western Blotting Detection System (GE Healthcare).

Images were obtained with an ImageQuant LAS-4000 imaging system (GE Healthcare). Fiji/ImageJ (N.H.I) software was used to quantify band densitometry. The background was removed from the densitometry of all the proteins. The values obtained were normalised to the densitometry of Glyceraldehyde 3-phosphate dehydrogenase (GAPDH). The normalised protein expression ratio was calculated relative to its control for each membrane analysed.

In order to obtain activity levels of phosphorylated proteins, densitometry of phosphorylated protein was divided by densitometry of total protein. Percentages of protein expression were compared using Student’s t-test.

### Statistical analysis

All data were analysed and plotted with Graph-Pad PRISM 8.1.2 software (GraphPad Software, Inc., San Diego, CA, USA). Immunofluorescence-based experiments were carried out in three animals per genotype and western blot experiments were carried out in four to eight animals per genotype.

The normal distribution of data sets from immunofluorescence-based experiments was determined with Shapiro Wilk and D’Agostino Pearson normality tests. Depending on the result of the normality tests, we performed parametrical (Student’s t-test) and non-parametric analysis (Mann Whitney test to compare ranks and Kolmogorov-Smirnov test to test differences between distributions). Significance of the values between the means of the data from western blots was compared using unpaired two-tailed Student’s t-test when the variances resulted in equal or two-tailed Welch’s t-test when the variances resulted unequally. Data are presented as the mean ± standard error of the mean (sem). Significant P-values: *P ≤ 0.05; **P ≤ 0.01; ***P ≤ 0.001 were considered.

## Supplementary information

Supplementary information.
