# Supplementary material for: Cofilin dysregulation alters actin turnover in frataxin-deficient neurons
Source: Sci Rep. 2020 Mar 23;10:5207. doi: 10.1038/s41598-020-62050-7 (PMC7090085; doi:10.1038/s41598-020-62050-7)
Supplement: Supplementary file 1 — Supplementary information. [file 41598_2020_62050_MOESM1_ESM.pdf]

Cofilin dysregulation alters actin turnover in frataxin-deficient neurons

Diana C. Muñoz-Lasso[1,2,3], Belén Mollá[1,4], Pablo Calap-Quintana[1,2,3], José Luis García-Giménez[1,2,3], Federico V. Pallardó[1,2,3], Francesc Palau[1,5,6], Pilar Gonzalez-Cabo[1,2,3*]

[1] CIBER de Enfermedades Raras (CIBERER), Valencia, Spain

[2] Department of Physiology, Faculty of Medicine and Dentistry. University of Valencia-INCLIVA, Valencia 46010, Spain

[3] Associated Unit for Rare Diseases INCLIVA-CIPF, Valencia, Spain

[4] Instituto de Biomedicina de Valencia (IBV), CSIC, Valencia 46010, Spain

[5] Institut de Recerca Sant Joan de Déu and Department of Genetic & Molecular Medicine and IPER, Hospital Sant Joan de Déu, Barcelona 08950, Spain

[6] Hospital Clínic and Division of Pediatrics, University of Barcelona School of Medicine and Health Sciences, Barcelona, Spain

[*] corresponding author: Pilar González Cabo; pilargc@uv.es
Facultad de Medicina y Odontología
Avda. Blasco Ibañez, 1515
46010 Valencia, Spain.
Telf. 0034 963395036

Figure S1: **Frataxin-deficient neurons exhibit reduced neurite growth.**

(a) Images of immunofluorescence showing adult sensory neurons derived from YG8R and control mice (C57BL/6J). Neurons were cultured for 24 hours and stained with antibody against β-tubulin III (green). Squares (white) highlight growth cones in the distal part of growing neurites. Scale bar, 50 µm. **(b)** Violin plot shows the distribution of the values for neurite length in both genotypes (n=3 independent experiments, a total of 160 [control] and 128 [YG8R] neurons per genotype were assayed). Red horizontal bars indicate the median and black horizontal bars indicate interquartile. Data shows how most sensory neurons from YG8R mice extended shorter neurites (mean=224.9 ±11.33 µm) than those from WT mice (mean = 249.7 ± 10.75 µm). Kolmogorov-Smirnov test was used to analyse significant changes between genotypes (P = 0.0468).

Figure S2: Expression levels of proteins that regulate actin-dynamics in nerve roots of YG8R and control mice (C57BL/6J).

(a) Representative blots for the kinase LIMK1 (total) and its active form (p(Thr508)-LIMK1) and in nerve roots tissue of the YG8R mice. Quantitative analysis of the western blots shows non-significative changes in the ratio of active and inactive LIMK1 (n=10, P=0.939). (b-c) Representative blots and quantification for the actin-binding proteins, **(b)** Profilin-1 (PFN1), the Wiskott–Aldrich Syndrome protein (WASP), and (c) the Disheveled-associated activator of morphogenesis 1 (DAAM1). Quantitative analysis of western blots shows a tendency towards a higher expression of DAAM1 (n=8, P=0.211) in the nerve roots of YG8R mice compared with control mice (C57-BL/6J). Changes in the expression of PFN1 (n=8, P=0.913) and WASP (n=8, P=0.679) were not significantly different between the genotypes, and neither exhibited a tendency. Data are shown as a mean ± s.e.m. Significant changes between genotypes were assessed using Welch's t-test. *: P≤ 0.05; **: P≤ 0.01; ns: P>0.05. Full-length blots are presented in Figure S3.

Figure S3: Uncropped, full images of western blots.

Red-boxes mark the bands of interest and black boxes mark one gel. Figure numbers and proteins are shown with respective blots.

Figure S1

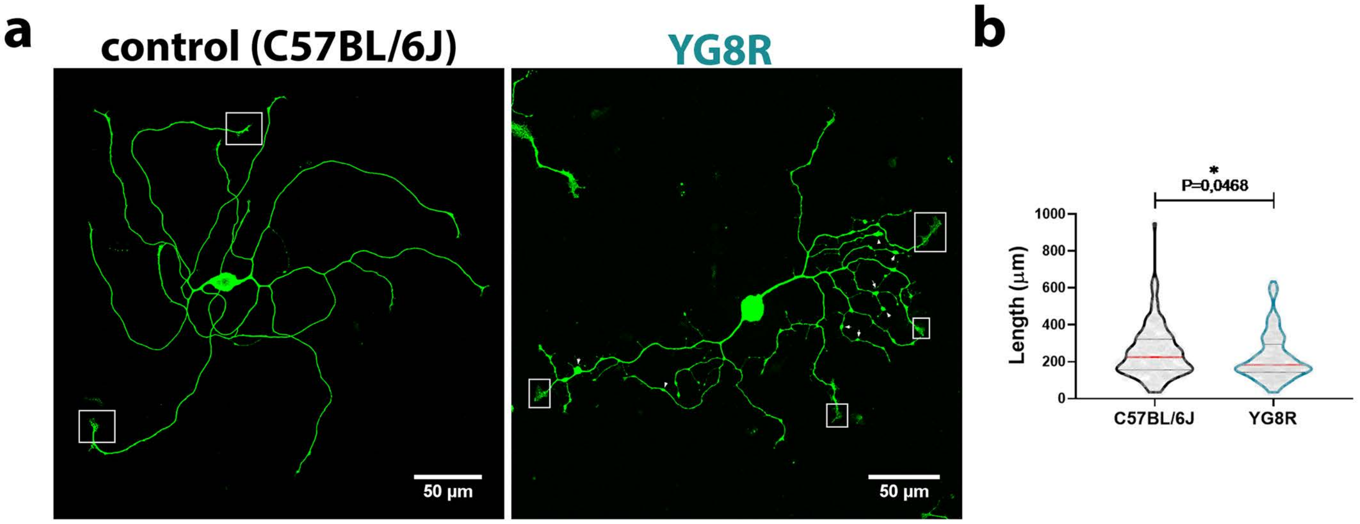
a
control (C57BL/6J)
YG8R
50 µm
50 µm
b
*
P=0,0468
Length (µm)
1000
800
600
400
200
0
C57BL/6J
YG8R

Figure S2

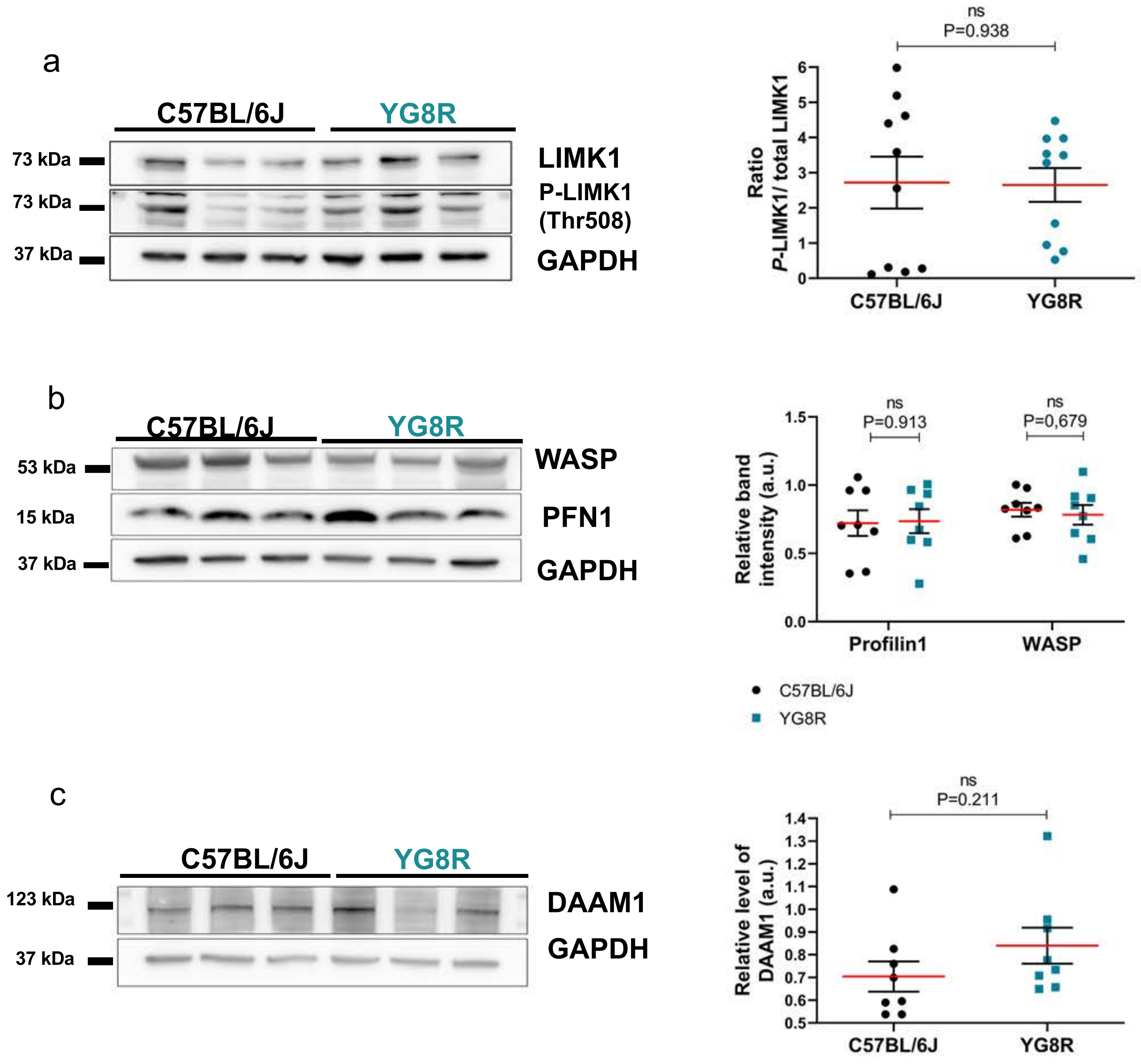

a
C57BL/6J
YG8R
73 kDa
73 kDa
37 kDa
LIMK1
P-LIMK1 (Thr508)
GAPDH
ns
P=0.938
Ratio P-LIMK1/ total LIMK1
C57BL/6J
YG8R
b
C57BL/6J
YG8R
53 kDa
15 kDa
37 kDa
WASP
PFN1
GAPDH
ns
P=0.913
ns
P=0,679
Relative band intensity (a.u.)
Profilin1
WASP
C57BL/6J
YG8R
c
C57BL/6J
YG8R
123 kDa
37 kDa
DAAM1
GAPDH
ns
P=0.211
Relative level of DAAM1 (a.u.)
C57BL/6J
YG8R

Figure S3

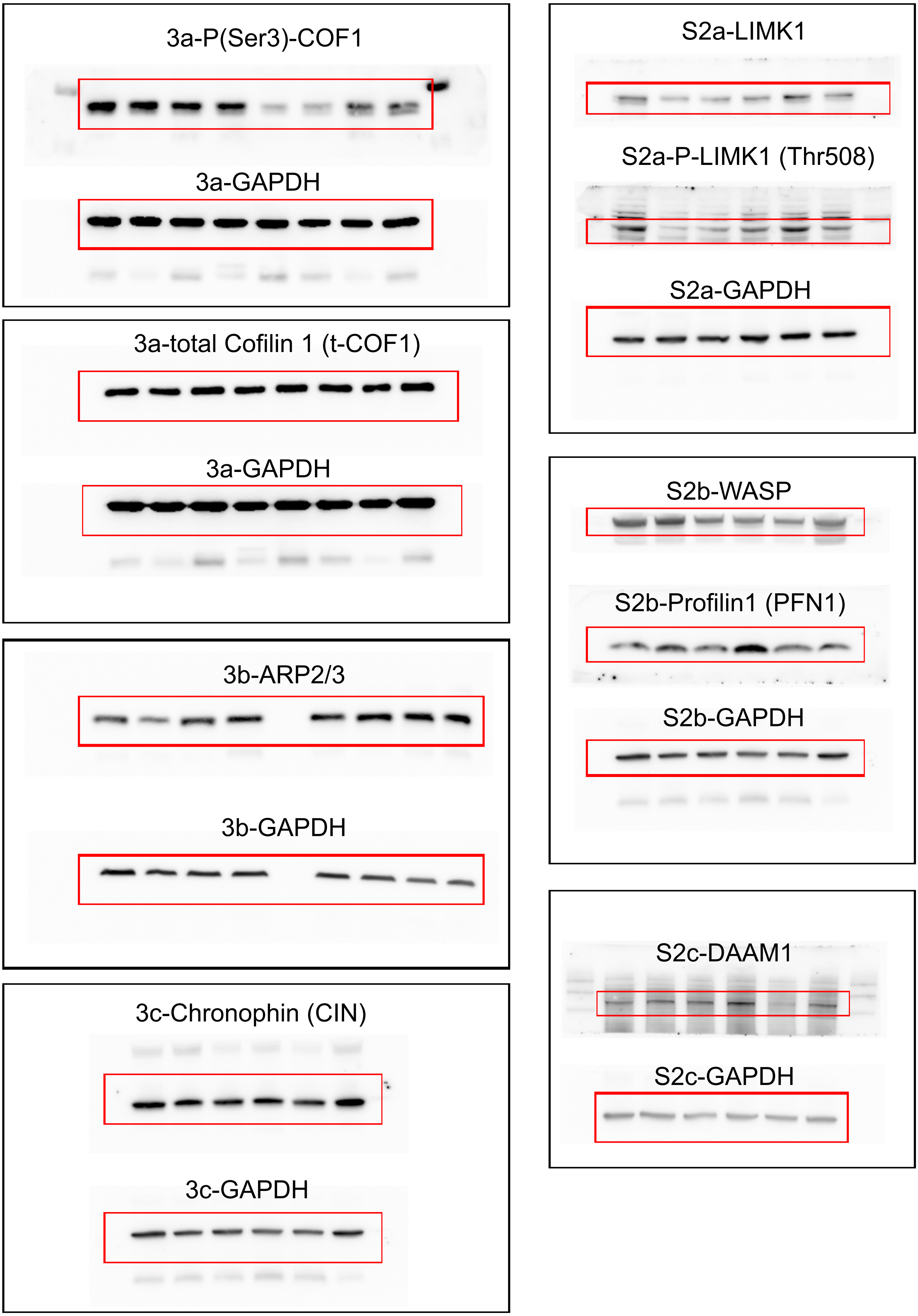
3a-P(Ser3)-COF1
3a-GAPDH
3a-total Cofilin 1 (t-COF1)
3a-GAPDH
3b-ARP2/3
3b-GAPDH
3c-Chronophin (CIN)
3c-GAPDH
S2a-LIMK1
S2a-P-LIMK1 (Thr508)
S2a-GAPDH
S2b-WASP
S2b-Profilin1 (PFN1)
S2b-GAPDH
S2c-DAAM1
S2c-GAPDH
